# Stigmasterol upregulates PDGFRα, contributing to white matter protection and anxiolytic-like behavior in a mouse model of vanadium-induced demyelination

**DOI:** 10.3389/fneur.2026.1706706

**Published:** 2026-01-30

**Authors:** Mohammad-Amin Abdollahifar, Meira M. F. Machado, Esmin Unaran, Olamide E. Adebiyi

**Affiliations:** 1 Veterinary Biomedical Sciences, Western College of Veterinary Medicine, University of Saskatchewan, Saskatoon, SK, Canada; 2 Translational Cognitive Neuroscience Lab, Robarts Research Institute, Western University, London, ON, Canada; 3 Canada Centre for Functional and Metabolic Mapping, Robarts Research Institute, Western University, London, ON, Canada

**Keywords:** behavior, demyelination, myelin, oligodendrocytes, stigmasterol, vanadium

## Abstract

**Introduction:**

Demyelinating lesions, or plaques, can form around axons when mature oligodendrocytes are damaged, often because of viral infections, heavy metal toxicity, or autoimmune disorders. These lesions are associated with cognitive impairment, motor dysfunction, sensory deficits, and memory loss, and may contribute to the progression of neurodegenerative diseases. At present, no therapy exists for demyelinating disorders, and available treatments primarily slow the progression of myelin loss while cognitive and functional deficits persist.

**Materials and methods:**

In this study, we investigated the neuroprotective potential of stigmasterol in a vanadium-induced demyelination. Forty-eight C57BL/6 mice were randomly assigned to three groups and received either saline, vanadium, or vanadium plus stigmasterol for 4 weeks. Behavioral assessments included the elevated plus maze and open field test for anxiety-like behavior, the Barnes maze for learning and memory, and grip strength and rotarod tests for motor function. Immunofluorescence staining and Western blotting were used to evaluate markers of oligodendrocyte lineage (Olig2, PDGFR*α*), myelin integrity (MBP, MOG, electron microscopy), neuronal survival (NeuN), and glial activation, while inflammatory cytokines (TNF-α, IL-6) were quantified by ELISA.

**Results:**

Our results revealed that vanadium administration induced anxiety-like behavior, impaired behavioral flexibility, reduced motor strength and coordination, and was associated with loss of MBP and MOG expression, decreased PDGFR*α* and Olig2, and elevated glial activation and inflammatory cytokines. Remarkably, stigmasterol co-treatment ameliorated these behavioral deficits, preserved MBP and MOG expression, increased PDGFRα and Olig2 levels, and attenuated microglial and astrocytic activation along with TNF-α and IL-6 production.

**Conclusion:**

These findings suggest that stigmasterol confers neuroprotection by preserving oligodendrocyte lineage cells, enhancing PDGFRα-mediated precursor recruitment, and maintaining myelin integrity. By mitigating neuroinflammation and promoting remyelination, stigmasterol is a promising therapeutic candidate for metal-induced demyelinating disorders.

## 1 Introduction

Exposure to potentially toxic elements such as arsenic, vanadium, cadmium, and lead has been a public health concern globally (1, 2). Particularly, this exposure has been linked to several neurological disorders such as multiple sclerosis, Alzheimer’s disease, amyotrophic lateral sclerosis, Parkinson’s disease, etc. (3–5). Disturbingly, animal and human exposure to these elements is on the increase following industrialization (6, 7). Vanadium is an emerging environmental contaminant naturally found in water, soil, and air. While short-term exposure to low doses has been reported to have antidiabetic, antitumor, and antiparasitic properties, long-term exposure to toxic levels can occur, particularly during fossil fuel combustion and in the steel and mining industries (38). Vanadium intoxication has been reported in industrial workers following inhalation of vanadium pentoxide dust, resulting in respiratory, gastrointestinal, and neurological symptoms (39, 40). Reports have documented that vanadium crosses the blood–brain barrier and accumulates in the brain, causing neuroinflammation that results in damage to myelin, hippocampal, cerebellar Purkinje neurons, and demyelinating lesions (8–10, 41).

Previously, we also showed in rodent models that this metal leads to altered behavior, impairment in spatial memory, and motor incoordination (8, 11). Furthermore, low-dose intranasal exposure to vanadium impairs olfactory behavior and causes damage to dopaminergic neurons in the glomerular layer of the olfactory bulb (12). In humans, neurotoxicity has become an increasing concern for industrial workers exposed to metal-containing substances, as well as residents and non-workers in the surrounding environments (42, 43). We demonstrated that one possible mechanism of vanadium-induced neurotoxicity may be driven by its ability to trigger oxidative stress, resulting in excessive production of reactive oxygen species (ROS). When ROS levels exceed the capacity of *in vivo* antioxidant enzymes, this results in lipid peroxidation and neurodegeneration (13). We have been investigating compounds that can mitigate these in the central nervous system. Several reports have demonstrated the neuroprotective potential of stigmasterol in models of neurological disorders and in heavy metal-induced neurotoxicity (8, 13–17). However, the mechanisms underlying its myelin-protective effects remain poorly understood. This study aims to elucidate the molecular basis of stigmasterol’s neuroprotective action in a model of vanadium-induced demyelination. Specifically, we investigate how stigmasterol promotes accelerated remyelination, improves behavioral and motor coordination outcomes, and modulates the expression of neuroinflammatory cytokines and myelin-associated proteins.

## 2 Materials and methods

### 2.1 Experimental subjects

Forty-eight male and female wildtype C57/BL6 mice (8-week-old, weight 20.08 ± 1.36 g) were obtained from the Jackson Laboratories (strain #000664, Bar Harbor, ME). They were randomly assigned to three groups of 16 mice/group (⧲ = 8, ⧬ = 8). The group sample size was determined based on a power analysis with 80% power and a 95% confidence interval to detect significant effects, while accounting for potential sex differences in treatment response. The first group received saline (the vehicle, 0.02 mL/g ip.) and served as the control, the second group was given vanadium (sodium metavanadate, 5 mg/kg ip. SigmaAldrich #72060), and the third group was treated with both vanadium (sodium metavanadate, 5 mg/kg ip.) and stigmasterol (0.1 mg/kg oral gavage, SigmaAldrich #S2424). All drugs were given in a single daily dose for 28 consecutive days. All experiments complied with the ARRIVE and Canadian Council on Animal Care guidelines. The study was approved by the University Animal Care Committee (UACC, AUP#2021–082) and the Animal Research Ethics Board.

### 2.2 Behavioral analysis

Mice were housed in a pathogen-free animal house in groups of four per cage under controlled conditions of 22–23 °C temperature, 50 ± 10% humidity, and a 12-h light/dark cycle. Animals had ad libitum access to water and standard pelletized rodent diet (Teklad, #8640).

All behavioral experiments were conducted between 09:00 and 15:00 in a testing room separate from the animals’ housing room. The testing room was illuminated at 100 lux, and mice were first habituated to the room for at least 30 min before commencing the test each day. The experimenter was blinded to each mouse group assignment during the behavioral assessment. After each trial, the behavioral mazes/apparatus were cleaned with 70% ethanol to prevent a bias based on olfactory cues from other tested mice.

#### 2.2.1 Elevated plus maze

The elevated plus maze (EPM) consists of four arms, 2 adjacent open (33.5 × 7 × 0.5 cm) and closed (33.5 × 7× 20 cm) arms, and a central platform (13.5 cm × 10 cm). The apparatus was raised 50 cm from the floor. Each session lasted for 5 min. The test was recorded using a video camera attached to a computer. The number of entries (an entry is defined as when the four legs of the animal were inside an arm) into each arm, the time spent in each arm, and the distance traveled during the test were also recorded as previously described (18).

#### 2.2.2 Rotarod test

The rotarod test was used to assess balance and motor function across the groups. Mice were placed on a rotating rod (Omnitech Electronics, Columbus, OH) with an accelerating speed. The test started at 4 rpm, increasing at a rate of 20 rpm per minute, up to a maximum of 36 rpm. On the first day, mice underwent habituation with 10 trials to familiarize them with the apparatus. On the test day, each animal completed four trials, and the average time before falling (fall latency) was calculated. Additionally, the total distance traveled and the final speed reached were recorded. Each session lasted for 5 min.

#### 2.2.3 Forelimb grip strength test

A grip strength meter (MK-380CM/R; Muromachi Kikai Co., Ltd., Japan) was used to measure forelimb grip strength. Before each test, the gauge was reset to 0 g after stabilization. The mouse was then allowed to grasp the bar attached to the force gauge with its forelimbs. The experimenter gently and gradually pulled the mouse’s tail backward until it released its grip, at which point the peak pull force (grip strength) in grams was recorded by the digital force transducer. Since the speed of tail pulling can affect results, the procedure was performed at a consistent and sufficiently slow pace to allow mice to build resistance against the force (19). Trials were excluded if the mouse used only one forelimb or its hindlimbs, turned during the pull, or let go of the bar without resistance. Each mouse underwent five consecutive measurements at one-hour intervals. To account for variability in performance, such as improper grasping with both forepaws and other technical errors, a qualitative assessment (good vs. bad) was applied to each recording. The three best readings per mouse were averaged to obtain the final grip strength measurement.

#### 2.2.4 Barnes maze

The Barnes maze consists of a white circular platform (100 cm diameter) with 20 equally spaced holes (5 cm diameter) and a black escape box mounted under one of the holes. Four visual cues of different shapes and colors were placed on each side of the maze. A centrally mounted overhead camera recorded all mouse movements during the 3-min session.

The test was divided into 2 phases (acquisition and prob). The acquisition phase consists of a total of sixteen sessions (4 trials/day) over the first 4 days. Mice were placed in the center of the maze and allowed 3 min to locate the escape box. We randomized the location of the escape box across the test mice. If a mouse failed to find it within the allotted time, it was gently guided to the box and kept there for 1 min before returning to its home cage. On the fifth day, the probe trial was conducted, identical to the acquisition phase but without the escape box. Each mouse underwent one trial. The recorded parameters during the acquisition phase included:

*Escape latency*: the time taken for the mouse to enter the escape box with its entire body.
*Primary errors:* the number of incorrect holes checked before locating the target hole.
*Search strategy*: classified as spatial, serial, or random based on the first head poke behavior:
*Spatial*: Mice made four or fewer primary errors before reaching the escape hole.
*Serial*: Mice systematically searched holes along the maze periphery in a clockwise or counterclockwise pattern.
*Random*: All other search behaviors, including failure to enter the escape box within 3 min.
For the probe trial, we analyzed the time spent in the quadrant where the escape box was previously located.

**Figure 1:**
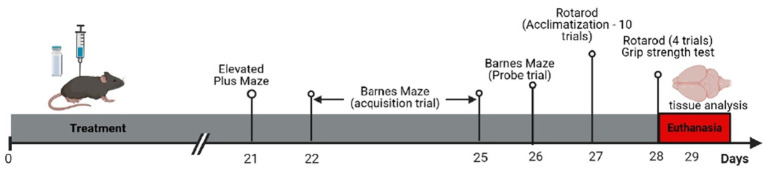
Experimental timeline; animals received either saline, vanadium (as sodium metavanadate), or a combination of vanadium and stigmasterol for 28 consecutive days before euthanasia. Behavioral testing began in the third week of substance administration.

All behavioral tests were recorded by a video camera fixed above the testing mazes. The videos were analyzed by an experimenter blinded to the treatment group using the AnyMaze Software. The timeline for the behavioral tests is presented in Figure 1.

### 2.3 Enzyme-linked immunosorbent assay (ELISA)

We measured the secretory levels of IL-6 (Thermo Cat#88–7,105-22) and TNF-*α* (Thermo Cat#88–7,324-22) in brain homogenates of a cohort of mice (*n* = 6/group, 3 males, 3 females) using ELISA kits according to the manufacturer’s instructions. The absorbance was read at 450 nm using an ELISA reader. We determined cytokine concentrations by plotting OD450 values against standard concentrations and estimating sample values from the standard curve. The left hemisphere was used to quantify these cytokines levels.

### 2.4 Western blot assay

Immunoblotting was performed as previously described by Mahmood and Yang (20) using the right hemisphere from the same cohort of mice (*n* = 6/group, 3 males, 3 females) described above for ELISA. Proteins were separated on SDS-polyacrylamide gels (12% for MOG; 8% for PDGFR*α*) and blotted onto nitrocellulose membranes (8 cm × 6 cm) using the Biorad® turbo transfer system. Membranes were immediately transferred to a blocking buffer and incubated with gentle agitation for 1 h at room temperature. Primary antibodies (MOG; MAB5680MI, FisherScientific, PDGFR*α*; Ab203491, Abcam) were incubated overnight at 4 °C in 5% milk in TBS with 0.1% Tween 20. HRP-coupled secondary antibodies α-rabbit-HRP (#65–6,120 Invitrogen) or α-mouse-HRP (#31430, Invitrogen) (1:10000) were incubated in 5% milk in TBS with 0.1% Tween 20 for 1 h at RT. This was followed by three 5-min washes in TBST at room temperature and incubation in Clarity western ECL substrate chemiluminescent detection reagent (Bio-Rad) for 5 min prior to detection with a ChemiDoc MP imager. Quantification was performed with ImageJ (21) using actin protein as a loading control, and graphs were plotted using GraphPad Prism 10.4.

### 2.5 Immunofluorescence assay

Mice (*n* = 6/group, 3 males, 3 females) were anesthetized with a ketamine/xylazine cocktail and transcardially perfused with phosphate-buffered saline (PBS) followed by 4% paraformaldehyde (PFA). After cervical dislocation, brains were carefully dissected, post-fixed in 4% PFA overnight, and then transferred to 30% sucrose solution for cryoprotection. Brain tissues were coronally sectioned at 20 μm thickness using a cryostat. Free-floating sections were collected serially, with four consecutive sections placed into a single well of a 24-well plate and stored in cryoprotectant solution (PBS: ethylene glycol:glycerol, 2:1:1) at −20 °C until further processing. Sections corresponding to the hippocampal region were selected from sections 220–264 (well plates 55–66), corresponding to approximately Bregma −1.5 to −2.6 mm according to the Paxinos and Franklin mouse brain atlas (22). For immunostaining, brain sections were incubated with primary antibodies at 4 °C overnight. Sections were washed three times in PBS for 5 min each, then incubated with the appropriate secondary antibodies at room temperature for 1 h. After a final PBS rinse, sections were mounted using VECTASHIELD® Antifade Mounting Medium with DAPI (#H-1200-10). Images were acquired using a Leica microscope. Immunohistochemical quantification was conducted using ImageJ software (Fiji). The number of positive cells was counted within predefined regions of interest (ROIs) and results were reported as cells/μm^2^. In addition, the intensity was quantified by measuring the mean gray value after subtraction of background using identical threshold settings for all images. The complete list of antibodies used in this study is available in the Supplementary Table 1.

### 2.6 Transmission electron microscopy

#### 2.6.1 Perfusion and tissue preparation

Mice (*n* = 4/group, male = 2, female = 2) were perfused transcardially with 0.1 M phosphate buffer (PB; pH 7.4), followed by a fixative solution containing 4% paraformaldehyde (PFA), 2% glutaraldehyde, and 0.1 M PB. Following dissection, brains were post-fixed by immersion in 20 × the volume of the same fixative for 48 h at 4 °C. After fixation, tissues were rinsed extensively in 0.1 M PB (3–4 buffer changes over several hours). Coronal brain slices (100 μm) were sectioned using a vibratome and stored at 4 °C in 0.1 M PB until further processing.

#### 2.6.2 Embedding in epoxy resin

Slices were rinsed twice (10 min each) in 3% glutaraldehyde prepared in cold 0.2 M sodium cacodylate buffer. Post-fixation was performed in 2% osmium tetroxide (OsO₄) for 1–1.5 h at room temperature in a fume hood with vials protected from light. Samples were then washed in cold 0.2 M cacodylate buffer (2 × 10 min) and distilled water (2 × 5 min). Dehydration was carried out through a graded ethanol series (50, 70, 90%), followed by washing in propylene oxide (3 × 30 min at RT). Finally, tissues were infiltrated overnight with a 1:1 mixture of propylene oxide and epoxy embedding resin at room temperature with gentle agitation.

### 2.7 Statistical analysis

Statistical analysis was performed using GraphPad Prism software 10.4.1. Kolmogorov–Smirnov test was done to determine the data normality. We then used the one-way analysis of variance (ANOVA) to determine the significance between the experimental groups, followed by Tukey’s multiple comparisons post-hoc test to determine whether specific pairs of groups are significantly different from each other. Data are presented as Mean ± Standard Error of Mean, the differences with *p*-values ≤ 0.05 were considered statistically significant.

## 3 Results

### 3.1 Stigmasterol administration mitigates the anxiogenic effect of vanadium

**Figure 2:**
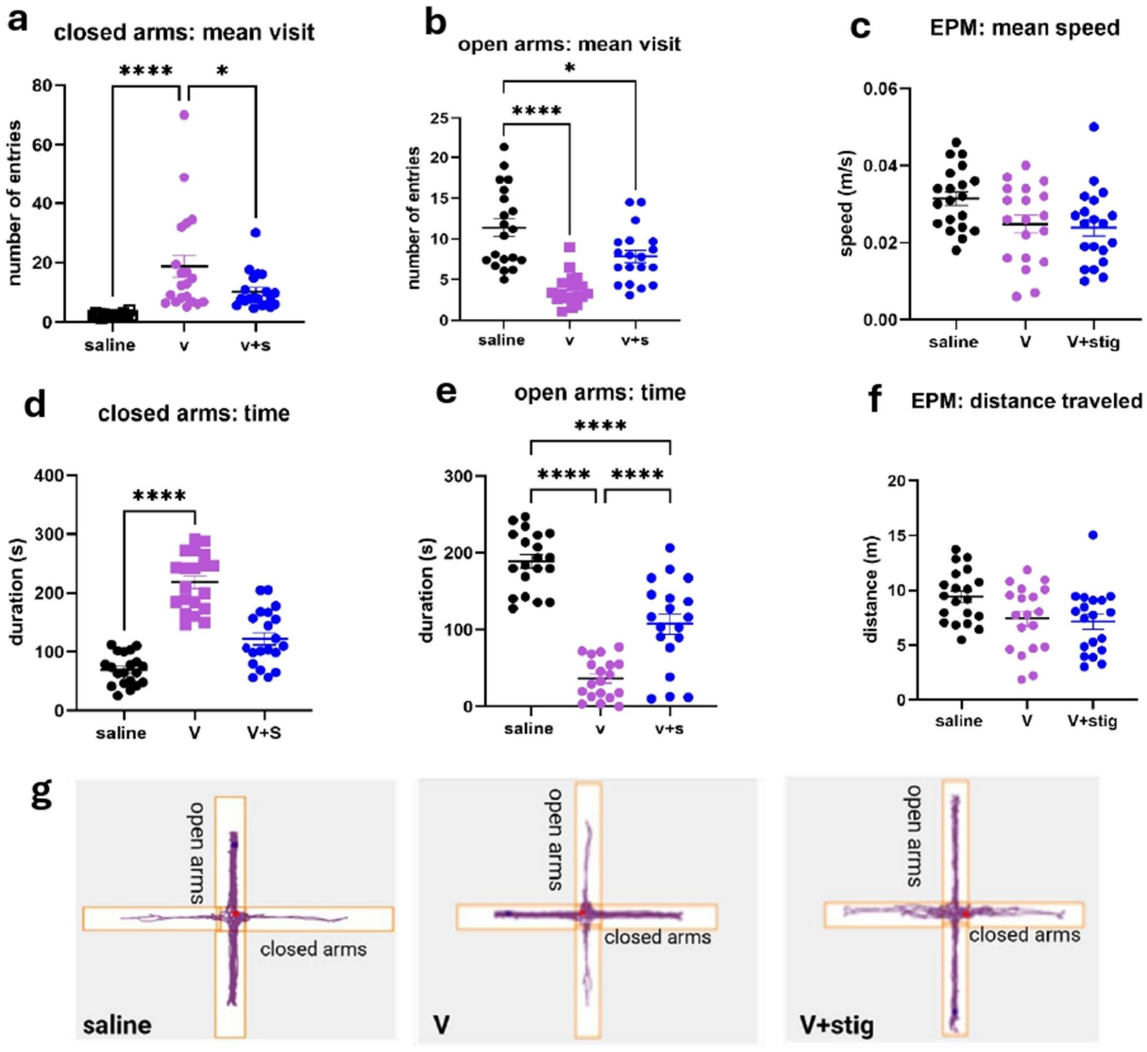
Elevated plus maze tests were conducted to assess anxiety-like behavior in C57BL/6 mice subjected to vanadium-induced neurotoxicity and treated with stigmasterol. **(a)** number of closed arm entries; **(b)** number of open arm entries; **(c)** mean speed (m/s); **(d)** time spent in closed arms (s); **(e)** time spent in open arms (s); **(f)** distance traveled (m); **(g)** representative spatial track of mice location during the elevated-plus maze test. Data were analyzed using one-way ANOVA followed by Tukey’s *post hoc* multiple comparisons test. ^*^p <0.05; ^****^p <0.0001 indicates statistical significance between study groups.

Our results indicate that mice administered vanadium spent significantly more time in the closed arms of the elevated plus maze (EPM) compared to the control group (*F*_(2,25)_ = 12.57; *p* < 0.0001) (Figure 2). Additionally, the time spent in the open arms was significantly lower in the vanadium-treated (V) group. However, while the vanadium plus stigmasterol (V + Stig) group also spent less time in the open arms compared to the control, their open-arm duration was significantly higher than that of the V group (*F*_(2,55)_ = 62.89; *p* < 0.0001). Similarly, the number of entries to the closed arm of the maze was significantly higher in the vanadium group compared to the control and vanadium+stigmasterol group (V vs. saline, *p* < 0.0001; V vs. V + stig, *p* = 0.0421, Figure 2a). Likewise, the mice in the vanadium group had significantly lower entries into the open arms of the EPM when compared with the control (V vs. saline, *p* < 0.0001, Figure 2b). These findings suggest that vanadium treatment increases anxiety-like behavior, as indicated by greater time spent in the closed arms, whereas co-administration with stigmasterol partially mitigates this effect. To determine whether these behavioral differences were due to variations in locomotor activity, we analyzed the total distance traveled and mean speed within the EPM. No significant differences were observed across groups, indicating that the effects of vanadium on anxiety-related behavior are unlikely to be confounded by alterations in locomotion.

### 3.2 Treatment with stigmasterol improved motor function in V-treated mice

**Figure 3:**
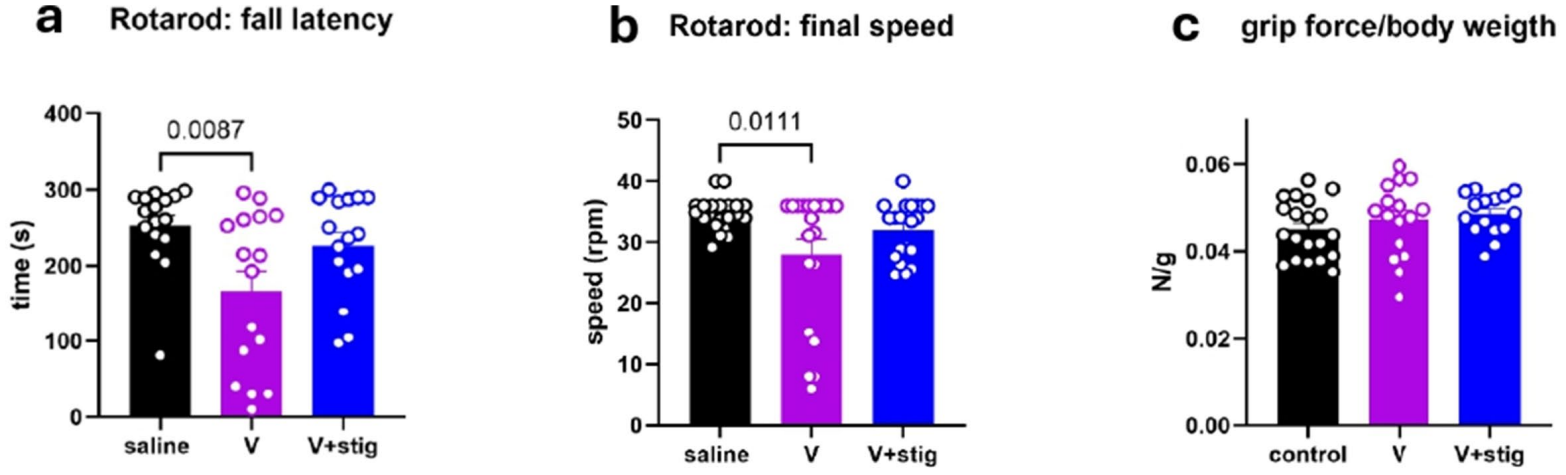
Rotarod and forelimb grip strength tests were conducted to assess motor coordination and forelimb strength, respectively. Data represent the mean performance across four trials per mouse: **(a)** duration on before falling off the rod, **(b)** maximal speed at the time of fall, **(c)** forelimb grip force per body weight. Data are expressed as mean ± SEM. Statistical evaluations were done by the one-way ANOVA followed by Tukey’s post hoc multiple comparisons test. Corresponding *p*-values are shown on the graphs.

To investigate the role of stigmasterol in motor function following V-induced toxicity, we used the rotarod test to evaluate motor coordination and the forelimb grip strength test to measure grasping ability and forelimb muscle strength. After 28 days of drug administration, V-treated mice exhibited the shortest fall latency on the rotarod, indicating impaired motor function. The V + Stig group showed an intermediate fall latency. Statistical analysis revealed a significant reduction in fall latency in the V-treated group compared to the control group (*p* = 0.0087, Figure 3a). However, the fall latency in the V + Stig group was comparable to that of the control group (*p* = 0.6136, Figure 3a), suggesting a protective effect of stigmasterol. A similar trend was observed in the final speed at which V-treated mice fell from the rotarod compared to controls (Figure 3b). We also assessed grip strength across treatment groups but found no significant differences between the V-treated and other groups (Figure 3c, *p* = 0.9272 vs. saline, *p* = 0.6483 vs. V + stig). To account for potential confounding effects of body weight, we analyzed grip force relative to body weight and still found no statistically significant differences.

### 3.3 Vanadium-treated mice exhibited intact learning and memory but relied on a serial search strategy

**Figure 4:**
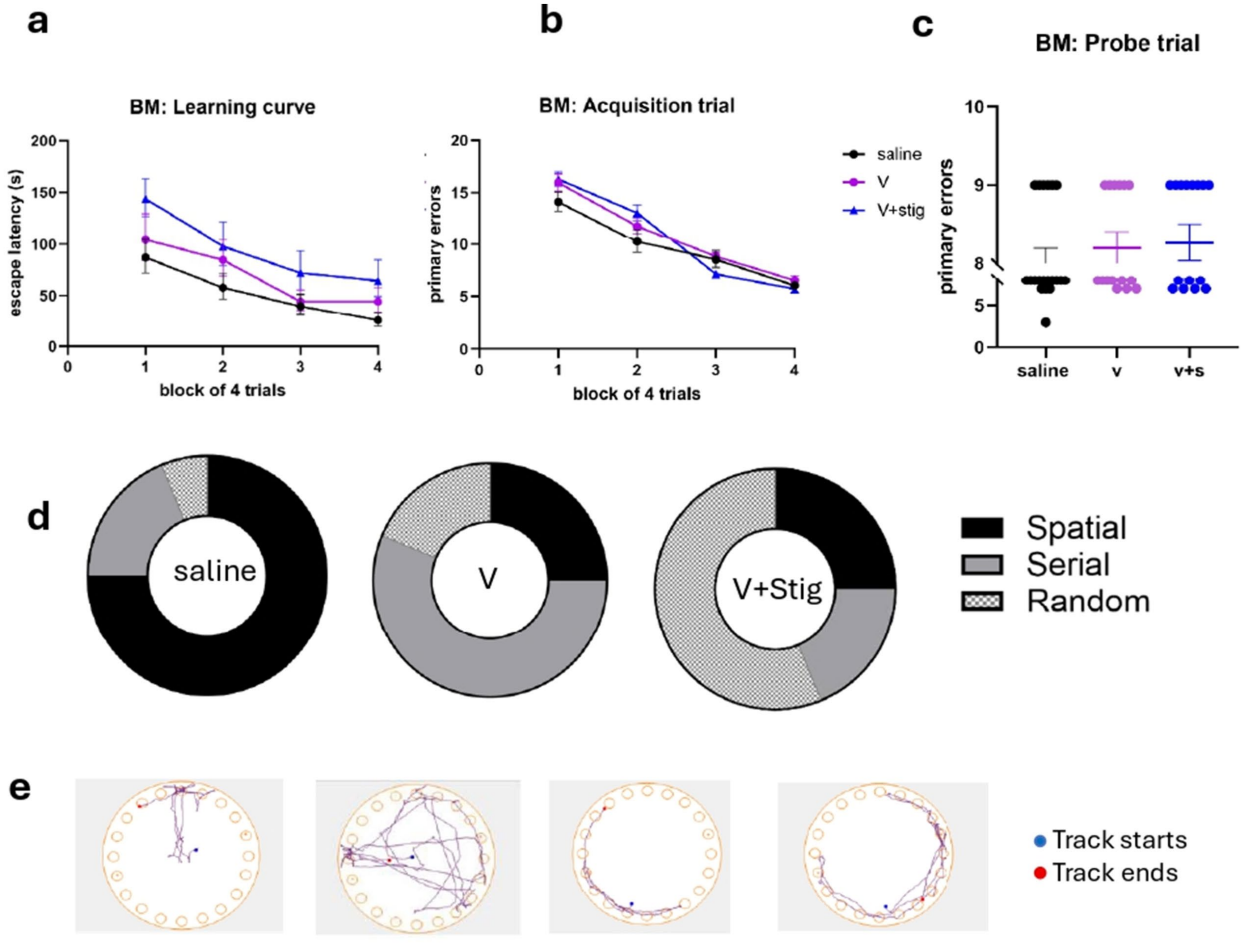
Learning curves and primary errors in the Barnes maze test. **(a)** Learning curve across acquisition trials. **(b)** Number of primary errors during acquisition trials. Each block on the *x*-axis represents the mean of four trials conducted per day over four consecutive acquisition days. **(c)** Number of primary errors during the probe trial (single trial on day 5). **(d)** Search strategies used by mice during the probe trial. **(e)** Track plot showing the position of the center of the animal during the test. Spatial, random, serial, serial strategy, respectively. Data from the acquisition trials were analyzed using a two-way repeated-measures ANOVA, whereas data from the probe trial were analyzed using a one-way ANOVA followed by Tukey’s *post hoc* multiple comparisons test.

Hippocampal-dependent learning and spatial memory were examined using the Barnes maze test between days 22 and 26 of drug administration (Figure 4). During the acquisition phase of the task, all three experimental groups showed significant learning, as revealed by a decrease in the escape latency with increasing number of trials (Figure 4a). For the escape latency, there was no significant interaction (two-way repeated measures analysis of variance, (RM-ANOVA), = *F*(2, 177) = 4.91 *p* = 0.8913 *n* = 180) but a main effect of acquisition day (two-way RM ANOVA, *F* (177, 531) = 1.361 *p* < 0.0001, *n* = 180) and treatment (two-way RM ANOVA, *F* (2.674, 473.3) = 526.6, *p* < 0.0001 *n* = 180), with V + stig mice performing significantly worse than both saline and V mice on day 1 (Tukey’s multiple comparison post-hoc analysis, *p* < 0.0001, Figure 4a). By day 4, the escape latency was found to be significant between the V and V + stig when compared with saline (*p* = 0.0270 saline vs. V, *p* = <0.0001 saline vs. V + stig). For primary error, there was no significant interaction (*p* = 0.6889 saline vs. V, *p* = 0.5745 saline vs. V + stig, *p* = 0.9830 V vs. V + stig). Mice were tested for spatial memory performance during probe trials carried out on day 5 (1 day after the final acquisition day) (Figure 4b).

We observed that the mice initially used non-spatial strategies to locate the hidden platform, but as training proceeded and they acquired information about the spatial location of the platform, they increasingly used more cognitively demanding strategies that are based on spatial cue configuration. Not surprisingly, all mice were equally efficient, during the acquisition trials, in this gradual deployment of spatial search strategies. However, during the probe trial, when the escape box was removed, only the saline mice were able to continue and adapt their use of mostly spatial strategies (saline = 75% V = 25% vs. V + s = 18.75% Figure 4c). We observed that the V mice largely failed to deploy spatial strategies during the probe but used more of a serial strategy, while the V + stig mice relied more on a random strategy (Figure 4d). Post-hoc comparison of overall strategies used during the Barnes maze test for spatial memory demonstrated that V mice deploy significantly fewer spatial strategies compared to saline and significantly higher random strategies compared to control mice. The track plots showed the movement paths of animals during each trial, highlighting exploration patterns to locating the target escape hole (Figure 4e).

Taken together, the probe trial data suggest that while the overall learning patterns were comparable across groups, differences emerged in the strategies used to locate the escape box.

The animals were monitored for weight gain/loss during the experiment; no changes were observed across the groups (data not shown).

### 3.4 Stigmasterol supplementation modulates vanadium-induced inflammatory response

**Figure 5:**
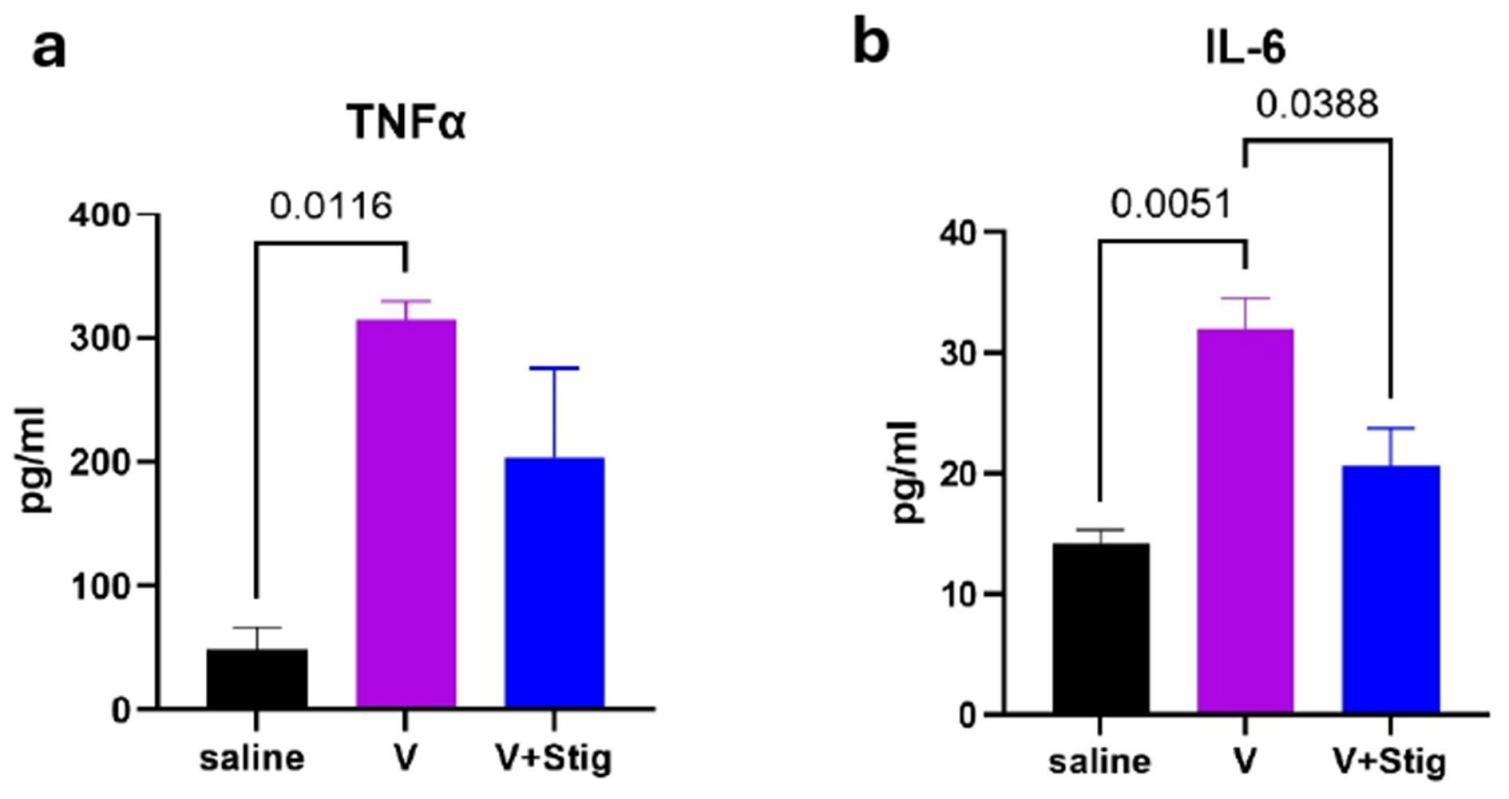
Brain levels of **(a)** TNFα and **(b)** IL-6 were assessed for pro-inflammatory cytokines. One-way ANOVA revealed significant differences between saline and V groups, but there were no significant differences between the V + Stig group and other groups in IL-6 and TNF-α concentrations. Corresponding *p*-values are shown on the graphs.

Our results revealed that exposure to vanadium significantly increased TNF-*α* (*p* = 0.0116) and IL-6 (*p* = 0.0051) (Figures 5a,b) levels in the brain compared with the saline group. The level of these pro-inflammatory cytokines in mice treated with stigmasterol (V + stig) was not different from the control (saline) group (TNF-α, *p* = 0.0993, IL-6 *p* = 0.2268). Further, the V + stig decreased significantly the level of IL-6 when compared to V (*p* = 0.0388) indicating a positive modulation of vanadium-induced elevation of IL-6. However, the levels of TNF-α although reduced in the V + Stig compared with the V group, this reduction was however not statistically significant.

### 3.5 Stigmasterol administration mitigates vanadium-induced neuroinflammation and enhances hippocampal remyelination by promoting the differentiation of oligodendrocyte precursor cells

**Figure 6:**
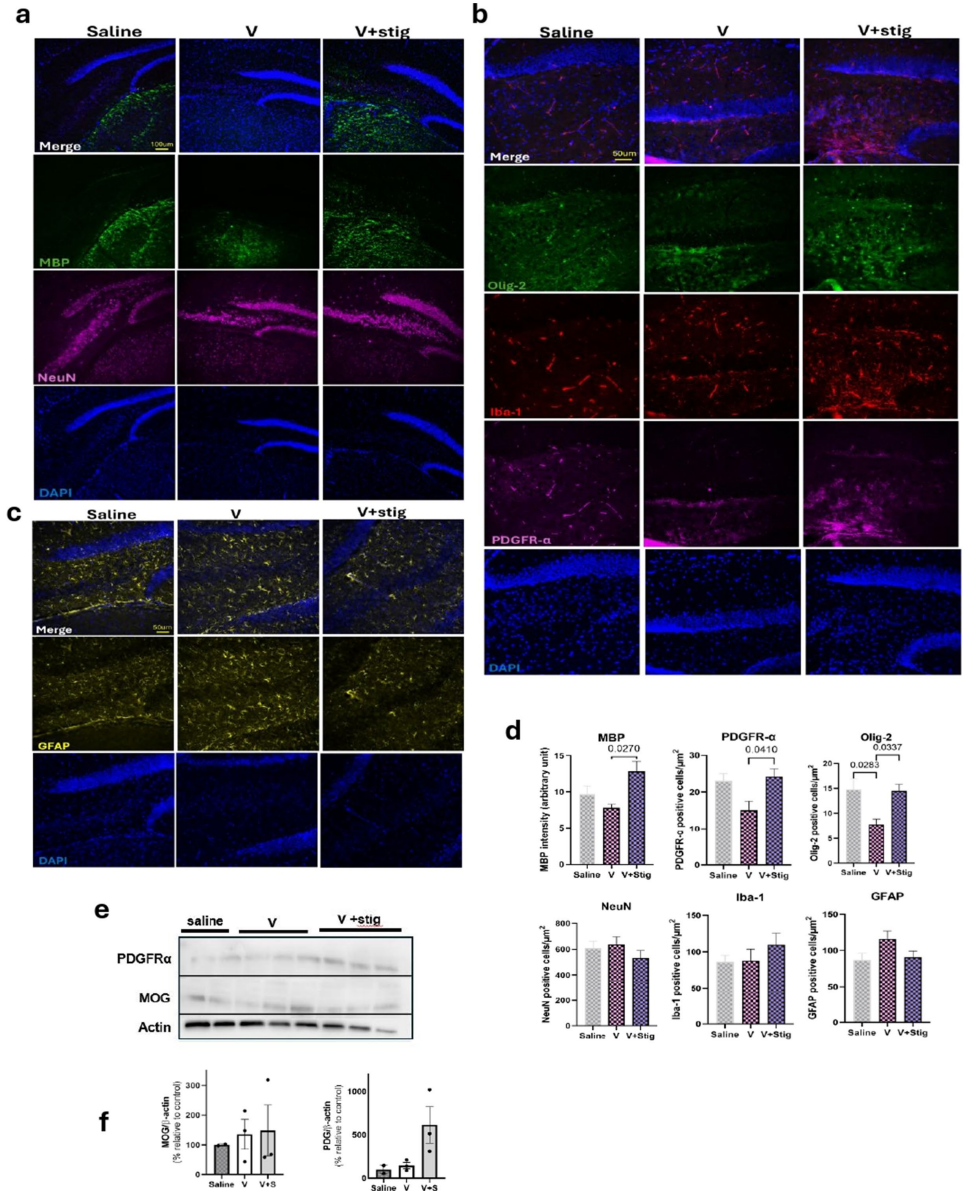
Immunofluorescence showing the expression of neuronal markers, myelin protein, glial expression, and PDGFRα in the hippocampal hilus around the dentate gyrus. **(a)** Double staining showing neuronal population (NeuN), saline V Ves expression of myelin basic protein (MBP), and nuclei are counterstained with DAPI (blue). **(b)** Triple staining showing the expression of the transcription factor Olig-2, microglial marker (Iba-1), and oligodendrocyte precursor cells (PDGFRα). **(c)** Expression of astrocytes was identified with GFAP in hippocampal sections. **(d)** Quantification of the intensity of MBP, number of PDGFR, Olig-2, NeuN, Iba-1, and GFAP. Data were analyzed using one-way ANOVA followed by Tukey’s post hoc multiple comparisons test. **(e)** Western blot of brain tissue from the study groups shows the expression levels of PDGFR-a and MOG, actin is used as a loading control. **(f)** Quantification of PDGFR-a and MOG protein band intensities across the treatment groups.

In the hippocampus (CA4), immunofluorescence staining revealed that vanadium exposure resulted in a significant downregulation of myelin basic protein (MBP) compared with the stigmasterol-treated group (*p* = 0.0270, V vs. V + Stig; Figures 6a,d). In addition, Olig2-positive cells were significantly reduced in the vanadium group, whereas co-administration of stigmasterol restored the expression of this transcription factor (*p* = 0.0283, V vs. Control; *p* = 0.337, V vs. V + Stig Figures 6b,d). A similar pattern was observed for PDGFRα expression (*p* = 0.410, V vs. V + Stig; Figures 6c,d). Although alterations in microglial activation (Iba-1), astrocytic response (GFAP), and neuronal density (NeuN) were observed, these changes did not reach statistical significance (Figures 6a–d). Collectively, these results suggest that stigmasterol exerts protective effects in this model by promoting myelin repair and preserving oligodendrocyte populations.

Western blot analysis of whole-brain lysates was performed to assess myelin oligodendrocyte glycoprotein (MOG) and PDGFRα levels, with actin used as the loading control (Figure 6e). Quantification revealed no significant differences across groups (Figure 6f).

### 3.6 Stigmasterol promotes spontaneous remyelination

**Figure 7:**
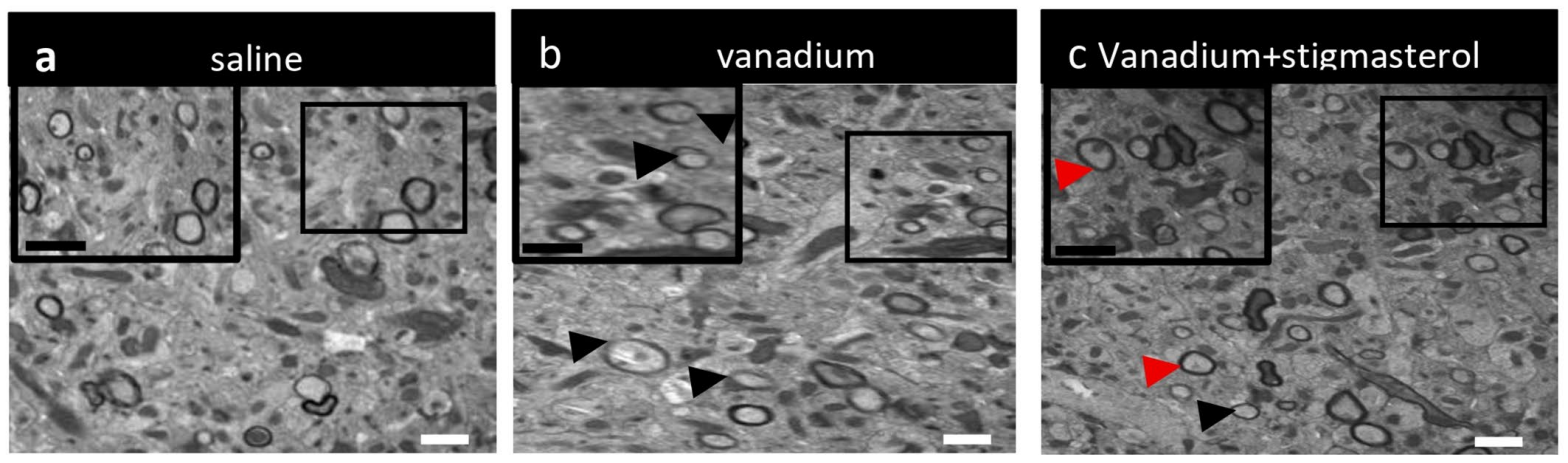
Representative transmission electron microscopy (TEM) images were captured from the hippocampal fimbria of **(a)** control, **(b)** vanadium group, showing several thinly myelinated axons (arrow heads), and **(c)** vanadium+stigmaasterol groups showing demyelinated (black arrowhead) and remyelinating (red arrowhead) axons. Scale bar: 1 μm, 5,800x.

The control group showed evidence of well-organized, uniformly myelinated axons with intact lamellar structure (Figure 7a). In contrast, the vanadium-treated group exhibited variably sized, densely packed axons with thin myelin sheaths, some displaying focal areas of lamellar splitting (Figure 7b). Concurrent administration of stigmasterol revealed signs of spontaneous remyelination, characterized by the presence of thin but continuous myelin sheaths relative to axonal diameter (Figure 7c).

## 4 Discussion

Exposure to vanadium has been shown to induce behavioral alterations, trigger neuroinflammation through the production of pro-inflammatory cytokines, and reduce endogenous antioxidant enzyme activity *in vivo*. Additionally, vanadium exposure has been associated with demyelination in specific brain regions. Building on our previous work, the present study aimed to further elucidate the mechanisms underlying stigmasterol’s myelin-protective potential. While our earlier study utilized stigmasterol directly isolated from *Grewia carpinifolia*, the current investigation employed a commercially available prototype of the compound to evaluate its efficacy in modulating neuroinflammatory pathways and preserving myelin integrity following vanadium exposure (8).

Our findings demonstrate that vanadium exposure induces significant behavioral disturbances, including increased anxiety-like behaviors and impaired motor coordination. In the elevated plus maze, vanadium-treated mice spent more time in closed arms and less in open arms, consistent with previous reports that vanadium disrupts neuronal signaling and elevates oxidative stress, leading to heightened anxiety responses (10, 13, 23). Co-treatment with stigmasterol partially mitigated these effects, suggesting an anxiolytic role for this phytosterol. Previous studies, including ours, have demonstrated that stigmasterol administration increases central zone preference, locomotion, and exploratory behavior in the open field test while simultaneously restoring antioxidant enzyme activity (8, 15). Consistent with these findings, our observation of increased open-arm preference in the stigmasterol-treated group mirrors this anxiolytic trend. However, a study by Panayotis et al. (24) using a chronic restraint stress model in mice reported no anxiolytic effect following a single 100 mg/kg dose of stigmasterol. This incongruity may be attributed to differences in dosing frequency, as repeated administration of phytosterol appears to yield more pronounced behavioral effects. Indeed, another study by Demonty et al. (25) demonstrated a tendency toward lower efficacy with single versus multiple daily doses of phytosterols, supporting the notion that repeated administration may be necessary to achieve optimal anxiolytic outcomes as observed in the present study.

The rotarod revealed that vanadium markedly impaired motor coordination, in line with earlier studies of vanadium neurotoxicity (26). In the current study, stigmasterol administration elevated fall latency, suggesting a protective effect on motor coordination, this improvement however, did not reach statistical significance compared with the vanadium group. Importantly, grip strength was not significantly altered, suggesting that vanadium predominantly affects coordination rather than muscular strength. Together, these results underscore stigmasterol’s ability to ameliorate vanadium-induced anxiety and motor impairments, likely through its antioxidant and anti-inflammatory properties. Stigmasterol has been reported to exert protective effects by suppressing microglial activation and attenuating inflammatory signaling (14, 27, 28). Specifically, it inhibits the AMPK–NF-κB/NLRP3 pathway, thereby reducing pro-inflammatory cytokine production *in vivo.* Consistent with these reports, our study demonstrated a significant downregulation of astrogliosis and neuroinflammatory cytokines following stigmasterol treatment. These results provide mechanistic support for the anxiolytic and motor-protective effects observed, suggesting that stigmasterol’s neuroprotective actions are mediated, at least in part, through the modulation of neuroinflammatory pathways.

Spatial learning and memory, assessed by the Barnes maze, revealed that all groups eventually learned the location of the escape box as the number of primary errors and escape latency was similar during the probe trial. However, differences were observed in the search strategies employed to find the escape box. These differences in strategy, despite similar escape latency, may reflect alterations in behavioral flexibility rather than overt memory impairment. Such findings suggest that vanadium exposure may subtly disrupt hippocampal-dependent cognitive processing without abolishing learning itself. Consistent with this finding, exposure to mixed metals including Cu, Co, Al, and Pb has been shown to impair cognitive flexibility in children, potentially through gut microbiota–mediated mechanisms (29), although evidence specifically implicating vanadium remains limited. Previous studies show stigmasterol exhibits both antioxidant and anti-inflammatory activities (17, 44), thus its beneficial effects on memory may be mediated by hippocampal modulation of oxidative stress and inflammation. Furthermore, stigmasterol has been reported to promote neuronal plasticity and upregulate immediate-early genes including Reln, Dcx, Egr1, and Ntrk2, which are important for memory consolidation (45). Nonetheless, mice from this group displayed a reduced tendency to shift toward spatial search strategy suggesting that while memory performance was preserved, flexibility in strategy may not have been fully restored. Given the absence of concurrent changes in anxiety-like behavior or locomotor activity, these findings should be interpreted with caution, as performance in the Barnes maze reflects a combination of cognitive, motivational, and exploratory processes rather than spatial memory and learning alone. Future studies incorporating complementary cognitive assays, such as the Y-maze and novel object recognition tests, will be necessary to more comprehensively evaluate the therapeutic potential of stigmasterol in mitigating vanadium-induced cognitive dysfunction.

Consistent with prior studies, vanadium exposure elevated brain levels of IL-6 and TNF-α, confirming its ability to drive neuroinflammation (46). Importantly, stigmasterol significantly attenuated these increases, restoring cytokine levels closer to control values. These findings align with evidence that phytosterols modulate neuroinflammatory pathways and may act as metabolic regulators in neurodegenerative conditions (47, 48). Our results showing that stigmasterol attenuates vanadium-induced neuroinflammation are in line with evidence from other disease models. For example, in Alzheimer’s disease models, stigmasterol has been reported to cross the blood–brain barrier, improve cognition, and suppress microglial-driven inflammation through AMPK-dependent inhibition of NF-κB and NLRP3 pathways (14). This suggests that the anti-inflammatory and neuroprotective actions of stigmasterol may extend across different pathological contexts, reinforcing its therapeutic potential. The ability of stigmasterol to suppress pro-inflammatory cytokines reinforces its role as a neuroprotective compound. This effect may be linked to its regulation of microglial activation and reduction of oxidative stress, mechanisms that require further exploration. Such cytokine modulation is clinically relevant, given the role of chronic inflammation in the progression of neurodegenerative disorders. Similarly, another study showed that stigmasterol shifted microglial polarization by suppressing pro-inflammatory (M1) markers and enhancing anti-inflammatory (M2) markers, both *in vivo* and *in vitro* (30). These effects were mediated through inhibition of the TLR4/NF-κB pathway, highlighting its role in promoting a protective microglial state. Although, vanadium exposure induced behavioral deficits and myelin-related alterations and the observed changes in NeuN, Iba-1 and GFAP were not statistical significantly different following stigmasterol concurrent administration. However, these findings could indicate that vanadium-induced neurotoxicity in the current is limited to white matter and oligodendrocyte pathology rather than severe neuronal loss or astrocytosis with stigmasterol’s protective effects targeting these myelin-related mechanisms.

Multiple brain regions are involved in regulating behaviors, including motor coordination and higher-order functions. Given the coordination and mild learning deficits observed in the vanadium-treated group, we examined potential structural alterations within the hippocampus. Immunofluorescence analysis revealed altered expression of myelin basic protein (MBP), a key marker of myelin integrity, in vanadium-treated mice. This finding aligns with previous reports linking heavy metal neurotoxicity to demyelination and white matter damage (49, 31). Remarkably, stigmasterol treatment preserved MBP expression and upregulated platelet-derived growth factor receptor alpha (PDGFRα), which is known to maintain the pool of oligodendrocyte precursor cells (OPCs) and promote their proliferation and survival (50, 51). The upregulation of PDGFRα suggests that stigmasterol not only prevents myelin loss but may also facilitate the replacement of damaged mature oligodendrocytes through enhanced OPC recruitment and differentiation. This regenerative mechanism is particularly relevant in the context of vanadium-induced neuropathology, where sustained demyelination contributes to long-term cognitive and motor deficits. By preserving oligodendrocyte function and promoting remyelination via PDGFRα signaling, stigmasterol demonstrates strong therapeutic potential for mitigating heavy metal–induced white matter injury.

White matter tracts within the hippocampus comprise its afferent and efferent connections and are therefore important for mediating hippocampal function. Among these, the fimbria represents a major white matter tract through which hippocampal outputs are transmitted to subcortical structures and the prefrontal cortex (32, 33). This tract facilitates the transmission of signals crucial for cognitive and spatial navigation (34, 35). Given its importance in cognitive flexibility and the significant differences observed in the search strategies employed by mice in the Barnes maze, we examined the microstructural integrity of the fimbria. Previous work demonstrated that vanadium-induced demyelination in white matter tracts, including the corpus callosum, resulted in cognitive impairment (31). The present findings extend this evidence by showing a significant correlation between fimbria microstructure and performance in cognitive tasks that specifically depend on hippocampal-prefrontal connectivity, consistent with earlier reports (36, 37). We also showed that demyelination in other hippocampal regions such as the hilus of the dentate gyrus hippocampal area is associated with cognitive impairments, while partial remyelination was observed following stigmasterol co-administration. This suggests that stigmasterol may mitigate vanadium-induced white matter pathology, at least to some extent. It is important to note that alterations in fimbria microstructure may also disrupt communication between the hippocampus and other interconnected regions not examined in this study, such as the nucleus accumbens and septum, which could in turn influence its connectivity with the prefrontal cortex (35) This consideration should be considered when interpreting our findings and designing future studies.

Taken together, our findings demonstrate that stigmasterol mitigates multiple aspects of vanadium-induced neurotoxicity, including anxiety, motor impairments, neuroinflammation, and myelin disruption, while only partially improving spatial learning strategies. These results underscore its potential as a therapeutic agent targeting demyelination and related neuropathologies. Future research should clarify dose-dependent efficacy, underlying mechanisms, particularly its regulation of oxidative stress, glial activation, and extend investigations to chronic exposure models and translational settings to establish clinical relevance.

## 5 Limitations of study

This study was not sufficiently powered to assess sex-specific differences, hence, data from male and female mice were pooled, and potential influences of sex hormones (including estrogen-related neuroprotection) could not be evaluated.

## Data Availability

The original contributions presented in the study are included in the article/Supplementary material, further inquiries can be directed to the corresponding author.
