# Supplementary material for: Stigmasterol upregulates PDGFRα, contributing to white matter protection and anxiolytic-like behavior in a mouse model of vanadium-induced demyelination
Source: Front Neurol. 2026 Jan 30;17:1706706. doi: 10.3389/fneur.2026.1706706 (PMC12903120; doi:10.3389/fneur.2026.1706706)
Supplement: Supplementary file 1 [file Table_1.docx]

**Table 1**: List of antibodies used

| **Antibody** | **Concentration** | **Catalog Number** | **Manufacturer** |
| --- | --- | --- | --- |
| **Primary antibodies** | | | |
| Proteolipid protein (PLP) | 1:1000 | 105784 | Abcam |
| Myelin basic protein | 1:500 | PA5-78397 | Invitrogen (Thermo Scientific) |
| GFAP | 1:500 | ab4674 | Invitrogen (Thermo Scientific) |
| PDGFRα | 1:200 | ab90967 | Abcam |
| NeuN | 1:5000 | PA5-143567 | Invitrogen (Thermo Scientific) |
| Olig-2 | 1:1000 | PA5-85734 | Invitrogen (Thermo Scientific) |
| **Secondary Antibodies** | | | |
| Goat anti-Rabbit IgG | Alexa Fluor™ 594 | A-11012 | Invitrogen (Thermo Scientific) |
| Goat anti-Mouse IgG | Alexa Fluor™ 488 | A-11005 | Invitrogen (Thermo Scientific) |
| Goat anti-Rat IgG | Alexa Fluor™ 647 | A48264 | Invitrogen (Thermo Scientific) |
| Goat anti-Chicken IgG | Alexa Fluor™ 647 | A32933 | Invitrogen (Thermo Scientific) |
